# Typhoid Fever

**Published:** 1878-07

**Authors:** J. R. Groover

**Affiliations:** Mica, Pickens Co., Ga.


### TYPHOID FEVER.

BY J. R. GROOVER, M.D., MICA, PICKENS CO., GA.

I propose briefly to give a plan for treating typhoid fever that has proved very successful in my hands, having used it in a great many cases during the past three years:

Diluted phos. acid 20 gtts. every four hours through the day, alternated with a powder composed of ipecac, camphor and prepared chalk, powder being changed as indications may require.

To allay nervous irritation and procure rest:

Bro. potassium every three hours through the night.

If the patient has more than three operations on the bowels in 24 hours, I usually prescribe tannic acid and opium, to be taken every time the bowels operate, until the diarrhœa is checked or the patient is thoroughly under the influence of the opium.

These remedies, with sponging the body and cold cloths to the head when the fever is high, and corn whisky through the night, if it is of the lower grade, with the necessary nourishment four times a day and once at night, will cure nearly every case of uncomplicated typhoid fever.

